# Evolution and development: engine-driven endodontic rotary nickel-titanium instruments

**DOI:** 10.1038/s41368-021-00154-0

**Published:** 2022-02-18

**Authors:** Yuhong Liang, Lin Yue

**Affiliations:** Department of Cariology and Endodontology, Peking University School and Hospital of Stomatology & National Clinical Research Center for Oral Diseases & National Engineering Research of Oral Biomaterials and Digital Medical Devices & Beijing Key Laboratory of Digital Stomatology, Beijing, China

**Keywords:** Endodontic files, Root canal treatment

## Abstract

Various engine-driven NiTi endodontic files have been indispensable and efficient tools in cleaning and shaping of root canals for practitioners. In this review, we introduce the relative terms and conceptions of NiTi file, including crystal phase composition, the design of the cutting part, types of separation. This review also analysis the main improvement and evolution of different generations of engine-driven nickel-titanium instruments in the past 20 years in the geometric design, manufacturing surface treatment such as electropolishing, thermal treatment, metallurgy. And the variety of motion modes of NiTi files to improve resistance to torsional failure were also discussed. Continuous advancements by the designers, provide better balance between shaping efficiency and resistance to of NiTi systems. In clinical practice an appropriate system should be selected based on the anatomy of the root canal, instrument characteristics, and operators’ experience.

## Introduction

Since Walia first reported the application of hand nickel-titanium (NiTi) files in clinical practice in 1988, various engine-driven NiTi files have been indispensable and efficient tools in cleaning and shaping of root canals for endodontic specialists.

**Fig. 1:**
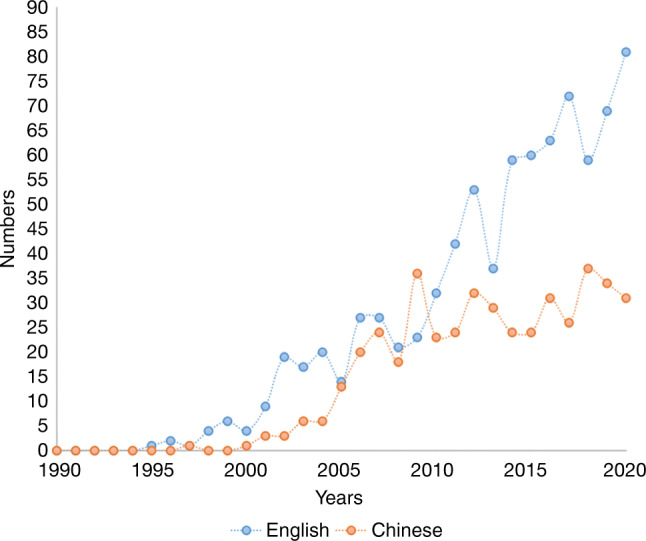
Chinese and English literature related to NiTi files, 1990–2020

Using “NiTi” and “NiTi file” as keywords, the literature from 1990 to 2020 was searched in MEDLINE, Scopus, Springer LINK, Annual Reviews, Cochrane, China National Knowledge Infrastructure (CNKI), Wanfang Data, and Chinese Scientific Journals Fulltext Database (CQVIP), which yielded 1275 papers, including 825 English papers and 450 Chinese papers. The inclusion criteria were as follows: the language was Chinese or English, and the full text was available. There are 300 studies in vivo and 975 studies in vitro. In the past 30 years the published papers had increased linearly and from 10 per year in 2000 to nearly 110 in 2020; with the twice growth in English compared with that of in Chinese (Fig. 1).

**Fig. 2:**
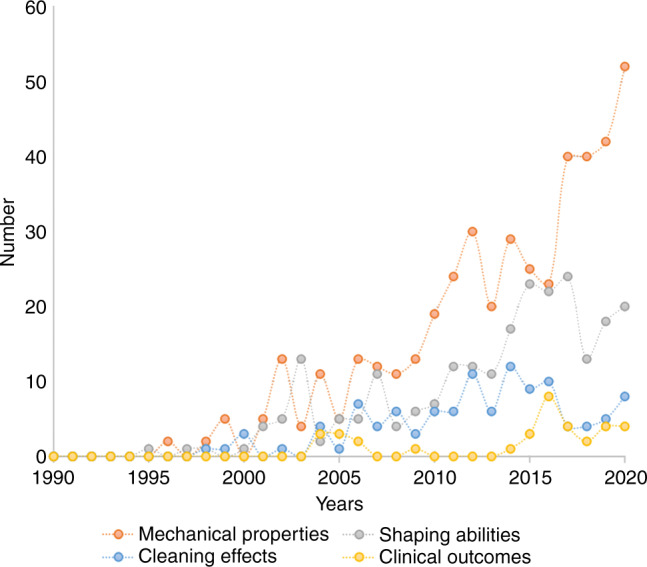
Distribution of English literature on NiTi files, 1990–2020

**Fig. 3:**
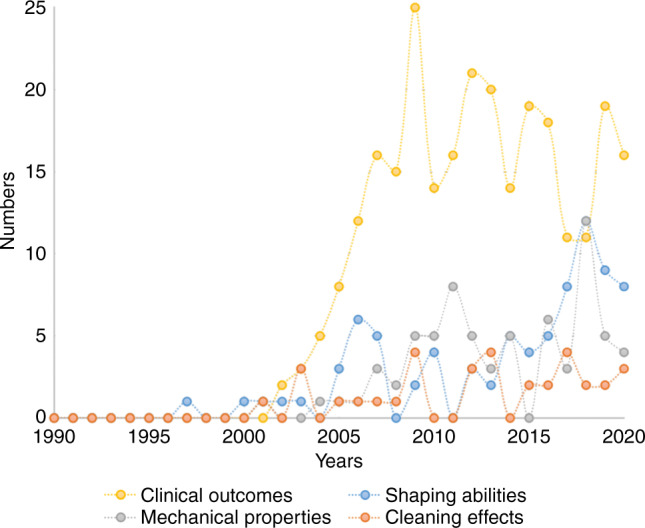
Distribution of Chinese literature on NiTi files, 1990–2020

These studies included 4 research directions: cleaning efficacy of instrumentation, shaping ability of root canal preparation, mechanical properties of NiTi files, and clinical outcome of root canal therapy. From 1995 to 2005, the number of studies had slowly increase. With the popularization of engine-driven rotary NiTi instruments in clinical practice, researchers continue to find and solve problems, and the technology, design, and manufacturing of NiTi instruments are constantly changing, thus promoting the rapid increase in research studies (Figs. 2, 3). From 2005 to 2021, more than 50% of the published studies, a total of 398 papers with an annual average of 27 papers, focused on the mechanical properties of NiTi files and ranking first among the topics. Mechanical properties of NiTi files cover the torsion, stiffness, and fatigue resistance of files and surface structure. The number of English studies on shaping ability ranks second. In the past 5 years from 2015 to 2021, the studies on efficacy of cleaning in English has increased slightly. But the English studies about clinical performance of NiTi files is relatively low, with a total of 35 papers. The number of Chinese papers increased rapidly during the 5 years from 2002 to 2007, including 265 clinical studies investigating quality of obturation, clinic emergency, and efficacy of cleaning. Research on the mechanical properties of NiTi instruments has been increasing since 2007.

Analysis of the literature composition shows that from the introduction of NiTi files to their implementation in clinical practice, the continuous improvement and evolution of NiTi files have always been centered on clinical problem-solving and treatment needs; that is, the core goal is to improve cutting efficiency and safety.

## NiTi files in dentistry

### Introduction and crystal phase composition of NiTi alloy

Nickel-titanium alloy was first successfully developed by the U.S. space program at the Naval Ordnance Laboratory in 1963 and was called nitinol (NiTi alloy). Due to its superelasticity (SE), shape-memory effect (SME), and biocompatibility, NiTi alloy was used to fabricate orthodontic wires in 1971 (ref. ^1^) and is also known as shape memory alloy (SMA). In 1975, Civjan proposed the concept of fabrication of endodontic instruments using NiTi.^2^ In 1988, the first NiTi hand file (#15) was introduced with orthodontic wire.^3^ Then, in the 1990s, commercial rotary NiTi files became available, and John McSpadden and Johnson, known as the fathers founders of rotary NiTi files, developed a file with a taper of 0.02 in 1992 and the ProFile Orifice Shaper (Dentsply Sirona, USA) with tapers of 0.04 and 0.06, respectively, in 1994. To date, more than 160 rotary NiTi systems have been used in clinical practice. Unlike stainless steel instruments that follow a unified international standard, no unified standard has been established for the design of rotary NiTi systems, and tip diameters, tapers, and blade lengths.

Equiatomic NiTi alloy contains similar numbers of nickel and titanium atoms (55% nickel and 45% titanium) and can exist in three microstructural phases: austenite, martensite, and the R-phase. At a high temperature austenite is called the parent phase, martensite at a low temperature is called the daughter phase, and both are the main crystal structures of the NiTi alloy. The lattice crystal structure can be changed by temperature and stress. The transformation temperature from austenite to martensite is 16–31 °C, indicating that conventional NiTi alloy mainly exists in the form of austenite at room and body temperatures. Pseudomartensite can be formed in a very small range of temperature range, and its Young’s modulus of pseudomartensite is lower than that of austenite, which is also called the R-phase. R-phase NiTi alloy would be flexible. When temperature is heated, the martensite can return to austenite. The NiTi alloy in austenic state is relatively stiff and has shape memory, and in the martensitic phase is flexible and ductile and can be easily deformed. The twinned phase structure of martensite is characterized by energy absorption and a damping effect, which may cause NiTi alloy to resist fatigue and lose metal memory.

### Terms related to the design of NiTi files

The design of the cutting part of an engine-driven NiTi file includes the flute-like cutting edge, the depth of the flute, the tip, and cross-sectional shape, rake angles, radial lands.

#### Helical flute angle and pitch

The helical flute angle is formed by the cutting edge and a cross section taken perpendicular to the long axis of the file, the pitch is the distance between two cutting edges in the lateral view. The NiTi file generally has 5–15 flutes. The tips of most engine-driven NiTi instruments are blunt and noncutting tips to guide instruments to the point of low resistance to avoid ledge formation or lateral perforation.

Instruments with a constant helical flute angle and pitch tend to result in the retention of cutting debris in grooves, especially the coronal groove, which increases the risk of the instrument becoming embedded in the dentin wall. A decrease in the pitch and an increase in the number of flutes increase the working cutting edge length of a file, which improves the cutting efficiency of the instrument, increases bending stiffness, and reduces stress.^4^ Instruments with increased pitch allow for superior debris removal and flexibility, but the risk of torsional failure is increased due to the distribution of high stress.^5^ Changes in flutes have different effects on NiTi files with different cross-sectional shapes. Instruments with square cross-sections are the least affected, while instruments with rectangular cross-sections are the most affected by the number of flutes.

The distance from the tip of the cutting edge to the bottom of the debris removal flute is the depth of the flute. When the instrument cuts the root canal wall, the stress gradually increases from the cutting edge to the flute.^6^ Increasing the space of the flute can accelerate the discharge of dentin debris during preparation, but obvious stress may be concentrated near the deep flute, which increases the possibility of defects or separation of the instrument.^7^ The curvatures and depths of the cutting edges and flutes of instruments with various cross-sectional shapes are different. For example, the concave triangle flutes of profile instruments have a curvature of 0.43 mm, while the debris removal flutes with a triple helix structure of Hero-624 instruments have a curvature of 0.125 mm.

#### Cutting edge, rake angle, and radial land

**Fig. 4:**
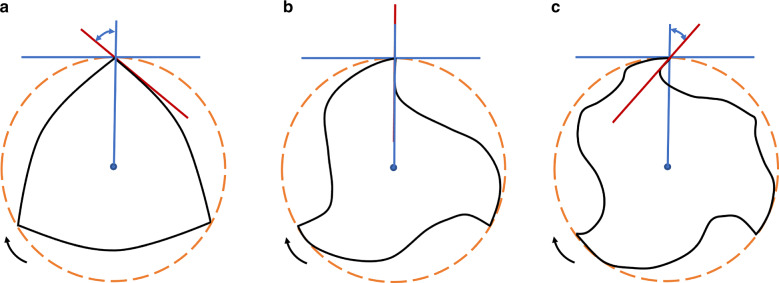
Diagrammatic representatives of rake angle cutting tip negative (**a**), neutral (**b**), and positive (**c**)

The rake angle is related to the slope and position of the cutting edge on the cross-section relative to the object, and the cutting angle of engine-driven NiTi files can be positive and negative. The rake angle is often depicted by drawing lines. The first line connects the cutting tip and the geometric center of the instrument, and the second line is tangential to the curve of the cutting face at its tip. When the first line is in front of the tangent line, the rake angle is positive; otherwise, it is negative (Fig. 4). Positive rake angle design allows for high cutting efficiency, but rapid wear. Instruments with a neutral or negative rake angle mainly grind the root canal wall and perform planning rather than cutting, which reduces the cutting efficiency of the dentin.^8^

The radial land is a surface located directly behind the cutting edge and is also called the radial supporting platform. The radial land imitates the shape of the tip to support the centric movement of the instrument in the root canal without causing canal transportation.^9^ A wide radial land increases peripheral strength of the instrument but this will lead to greater friction when cutting dentin;^7^ thus, a smear layer on the root canal wall easily forms.^10–12^ Except for Hero, early NiTi instruments generally adopt a radial land design.

#### Cross-sectional shape

Finite element (FE) analysis shows that the mechanical behavior of an instrument during the root canal shaping process is affected by the cross-sectional shape,^6,7^ include triangles, rectangles, or S-like quadrangles.^13^ For the same shape, an instrument with a larger cross-sectional area has higher torsional stiffness.^5^ Compared with the cross-sectional area, the area of the inner core is more related to the bending stiffness.^4^

#### Taper

The taper is the ratio of the diameter to the length of an instrument, which refers to the increase in diameter of the file per mm increase in length. Files with large and medium tapers are used for rapid cutting of dentin, and few changing times of files during instrumentation. Files with a small taper are conducive to the shaping of a good apical morphology and maintenance of the original axial direction of the root canal. Rotary NiTi files are usually designed with a large taper, and a section from the root canal orifice to the apical stop is prepared into a uniformity tapered funnel shape using the crown-down preparation technique.^9,14^ Most instruments are designed with a constant taper, i.e., the taper does not change on the same instrument. However, the systems produced by ProTaper Universal and ProTaper Next (Dentsply Sirona, USA) are characterized by a variable taper design, and the taper changes at different positions of the cutting edge of the same instrument.

### Separation and fracture of rotary NiTi files

Engine-driven NiTi files are efficient and convenient for root canal cleaning and shaping. However, safety is a major issue that must be considered in clinical practice since separation of NiTi instruments often occurs accidentally.^15^ The separation of NiTi files mainly occurs under two circumstances: flexural and torsional failures.^16^ Flexural failure occurs when the metal tolerance is exceeded, which is due to repeated compression and tension of the free-moving instrument at the curved root canal; thus, flexural failure is also commonly referred to as flexural cyclic fatigue.^15^ Torsional fracture occurs when the tips or any part of the instrument is locked in canal wall while the shaft continues to rotate by continuous apical driving force, due to the screw-in effect, which is known as torsional failure.^17^ Studies have shown that NiTi files with small inner core diameters tend to have more torsional failure, while files with large inner cores can resist torsional stress and are more prone to flexural failure.

The geometric morphology, manufacturing process, metallurgical development, and movement mode of NiTi files affect their performance of safety. Torsional stress and cyclic fatigue are applied to NiTi instruments in clinical practice and are also the risk factors determining the fracture and separation of NiTi instruments. To improve the flexural resistance of NiTi files, various file systems have been continuously improved and updated to increase their safety in clinical practice.

### Development and generation of NiTi files update

The NiTi file has been continuously developed in the more than 30 years since the first NiTi file came to market in 1992. In 2013, Haapasala reviewed the development of NiTi instruments and divided them into five generations. The first generation was represented by ProFile, LightSpeed (Lightspeed Inc, San Antonio, TX, USA), Quantec (Sybron Endo, CA, US), Greater Taper (Tulsa Dental Products, Tulsa, Okla), and Hero-642 (Micro-Mega, Besançon, France) in the 1990s. The main focus was the geometric design of a file, such as the cross-section and the shape of the flute, and safety was reflected by the design of the radial land for the cutting edge of the file. The second-generation was introduced from the late 1990s to the early 2000s, with representative instruments including ProTaper Universal, K3 (Sybron Endo, Orange County, CA, USA), Hero Shaper, BioRaCe (FKG Dentaire, La-Chaux-de Fonds, Switzerland), and EndoSequence (Brasseler, Savannah, GA, USA), and critical evolution focused on surface treatments such as electropolishing to reduce manufacturing defects and changes of geometric shapes such as the taper and rake angle to increase the cutting efficiency. Most second-generation files have no radial lands. In 2007, with the advent of martensite wire (M-wire) containing an increased percentage of martensite phase, NiTi files entered the third generation with a focus on material improvement. Subsequently, different materials, such as R-phase, controlled memory (CM)-wire, martensite-austenite electropolishing-flex-wire (max-wire), blue wire, and gold wire, were developed, and the fatigue resistance of NiTi files was continuously improved. Since 2008, based on the traditional continuous rotation mode, the risk of torsional failure in the fourth generation of NiTi files was reduced by improving the motion mode, such as reciprocating motion, combined motion, and axial motion. Single-file systems are also available at the same time, such as reciprocating single-file systems (Reciproc (VDW, Munich, Germany), WaveOne (Dentsply Maillefer, Ballaigues, Switzerland), twisted file adaptive (TFA) instruments (SybronEndo, Orange, CA, USA)), and a self-adjusting file (SAF) system (ReDent Nova, Ra’anana, Israel) with axial motion, and clinical efficiency was improved by reducing the number of preparation instruments. Since 2010, the eccentric rotary NiTi systems represented by ProTaper Next (Dentsply Sirona, US), Revo-S (Micro-Mega, Besançon, France), and OneShape (Micro-Mega, Besançon, Franc) have been regarded as the fifth generation.

**Fig. 5:**
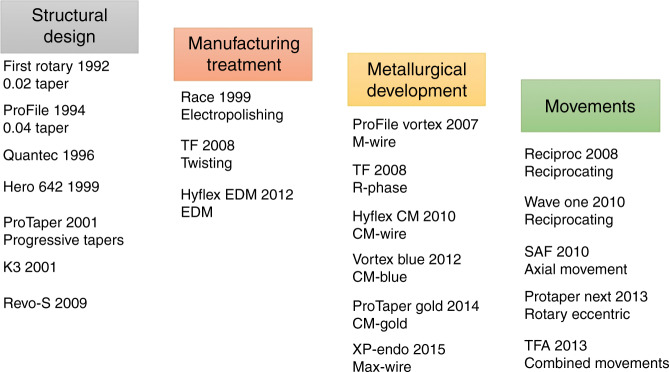
Development and representative systems of NiTi files

This review shows that the main improvements in NiTi files are concentrated in four aspects: flute design, manufacturing treatment, NiTi wire material, and motion mode. These improvements are arranged according to the occurrence time and have different emphases in different time periods, but they are complementary to each other and cannot be completely separated (Fig. 5).

## Evolution and improvement of NiTi files

More than 150 types of rotary NiTi systems are available for clinical applications. In the following section, engine-driven NiTi systems are summarized in terms of four aspects: structural design, metallurgical development, manufacturing treatment, and motion mode.

### Improvement and design of the geometric morphology of NiTi files

To obtain better clinical performance, an engine-driven NiTi file needs high torsional stiffness to improve the cutting efficiency and reduce the risk of flexural failure low bending stiffness to adapt to different root canal morphologies, which not only reduces the fatigue damage of the instrument but also avoids canal anatomy transportation. Changes to the geometric design and shape of NiTi files are applied in the cross-sectional shape, depth of the flute, area of the inner core, and radial land.^7^

#### The cross-sectional shape

Cross-sectional shape is the focus of improvement and optimization of several generations of NiTi files. The ProTaper Universal system uses a convex triangular cross-section design, which results in a more even stress distribution along the long axis of the instrument, a better cutting force, and a reduced risk of instrument separation.^18,19^ The Mtwo system (VDW, Munich, Germany) uses an “s”-shaped cross-sectional design with two cutting edges. The cutting edges are sharp, and the flutes between the edges are low and deep, which enhances the cutting efficacy and flexibility.^4^ Studies have found that Mtwo and Quantec instruments with asymmetric cross-sections have different inertias on the *x*-axis and *y*-axis. Therefore, the bending stiffness of the files in one direction is worse than that in the other direction, resulting in poor stability during root canal preparation and a tendency toward flexural failure. The cross-section of the ProTaper Next system is a rectangle, and it rotates eccentrically or asymmetrically, resulting in off-centered movement. This design improves the flexibility of the instrument, and asymmetric rotation reduces the screw-in effect and can reduce canal transportation.^20,21^ The cross-sectional design of the HyFlex EDM system has unique characteristics; that is, the cross-sectional shape along the cutting surface of the file is different. Near the handle of the file, the cross-section is triangular, providing better cutting efficiency; in the middle of the file, the cross-section is trapezoidal, providing stronger flexural resistance and better debris removal ability; at the tip of the file, the cross-section is quadrilateral, which can facilitate penetration of the instrument and reduce the risk of flexural failure.

#### Cutting edge, rake angle, and radial lands

Early systems such as Hero and K3 instruments, improve the cutting efficiency through a positive rake angle design, while ProFile, ProTaper Universal, Quantec, and other systems are designed with a neutral or negative rake angle, which reduces instrument embedding but affects the cutting efficiency (Fig. 4).^8^

#### Taper

Most engine-driven NiTi systems adopt the non-International Standardization Organization (ISO) design of a constant large taper; therefore, a set of files has the same taper but different tip sizes, which improves the cutting and shaping efficiency, and the stress is evenly distributed along the long axis, which reduces the risk of instrument failure. Exemplified as profile with a constant taper of 0.04. ProTaper files adopt a variable taper design, i.e., different tapers are designed for different parts of the same instrument, which considers both the flexibility of the instrument and cutting efficiency. ProTaper files can accurately complete root canal shaping according to the cutting target.^22^ The ProTaper Next system uses five files with different lengths and tip diameters for root canal preparation, i.e., X1 (17/0.04), X2 (25/0.06), X3 (30/0.07), X4 (40/0.06), and X5 (50/0.06). Among them, X1 and X2 are designed with increasing and decreasing tapers on a single file, respectively, and X3, X4, and X5 have fixed tapers starting from D1 to D3 and a gradually decreasing taper for the upward cutting part.^23^ The S-Apex instrument (FKG, La Chaux-de-Fonds, Switzerland) is designed with an inverted taper, which is conducive to establishing root canal patency in the apical area and allow weak areas at the junction of the working blade and handle; therefore, the removal of a fractured instrument is improved.

In addition, improvement in the helical flute angle and pitch can also improve the performance of an instrument. For the K3 XF instrument, the helical flute angle is designed to increase from the tip. From the tip to the crown, the pitch is increased, which may improve the debris removal ability of the flute and increase the safety of the instrument.^23^ The WaveOne single-file system uses two cross-sectional designs with different working lengths (D1-D8: modified triangle; D9-D16: convex triangle) and a reverse helix to vary the pitch and helical flute angle along the longitudinal axis, which increases the flexibility and safety.^24^

In summary, the geometric design of rotary NiTi files has been continuously developed to reach a balance between cutting efficiency and safety. For example, the ProTaper Next instrument, which adopts the two-point contact asymmetric rectangular cross-section design, has a large space for debris removal and a focus on high efficiency, while the Profile instrument has the characteristics of a negative rake angle, radial land, a noncutting guiding tip, and a constant taper, which highlights the safety considerations of ensuring centric movement of the instrument and reducing the embedding effect.

### Improvement in manufacturing or surface treatments

The manufacturing process affects the mechanical properties of NiTi instruments. Cutting by a precision lathe is the most frequently used processing method, which has been performed in the majority of NiTi instruments such as Profile, Lightspeed, ProTaper, Mtwo, and Hero. However, residual surface treatment defects, such as abscesses and cut marks, all become potential causes of instrument failure in clinical practice. The surface treatment techniques of twisting fabrication, electropolishing, and electrical discharge machining (EDM) have improved these problems to a certain extent, thus improving safety and reducing torsional failure.

#### Twisting fabrication

A conventional NiTi alloy is an SMA that can return to its original shape after deformation by an external force. When using twisting fabrication, which is used for stainless steel files, NiTi alloy wires cannot easily be formed and may be fractured in the process of twisting. After heat treatment, the NiTi alloy is transformed into the R-phase with lower shear modulus and can be manufactured with a twisting method. The Twisted File (TF) system is the representative instrument. Twisting fabrication avoids defects in the turning process of flutes, protects the crystal lattice structure of the metal from being damaged, and improves the fatigue resistance of the instrument. However, a strong screw-in effect can be produced during root canal preparation, leading to flares.

#### Electropolishing

The electropolishing process, also known as electrochemical surface treatment, refers to the deposition of metal ions on the surface of an object through electroplating to reduce surface irregularities in metal instruments. This method was first used to manufacture NiTi instruments by FKG in 1999. Mechanically turned NiTi instruments are immersed in an electrolyte bath to cause an oxidation-reduction reaction to form a uniform oxide layer on the surface of the instruments, which can reduce manufacturing defects and yield a smoother surface; therefore, increase the resistance to cyclic fatigue, cutting efficiency, and corrosion resistance of the instruments without affecting the superelasticity (SE). The Race, EndoSequence, and One Shape instruments are manufacturing using this technique. The TF system mentioned above also uses a special electrochemical surface treatment to enhance hardness and reduce surface irregularities while ensuring flexibility. Scanning electron microscopy (SEM) shows that the propagation of fatigue fractures of TF files after electropolishing treatment is curved, nonlinear, and independent of the surface structure. Such crack propagation is beneficial to improve the resistance to cyclic fatigue of TF files.^25^

#### EDM

EDM (electrical discharge machining) is a noncontact thermal erosion process widely used in engineering, which uses a controlled discharge process to melt the metal surface and evaporate small portions of the metal in the presence of a dielectric fluid and leaves a corroded rough metal surface. NiTi instruments manufactured using this treatment have a rougher and harder surface, thereby improving the cutting efficiency.^23,26^ In 2016, HyFlex EDM system (Coltene/Whaledent, Cuyahoga Falls, OH) manufactured using this spark-erosion technology was launched. X-ray diffraction analysis shows that the HyFlex EDM system is composed of martensitic NiTi alloy and substantial amounts of R-phase NiTi alloy, while the HyFlex CM system is composed of a mixture of martensitic and austenitic NiTi alloy.^27^ Hyflex EDM still has a higher hardness than conventional CM wires, despite absence of or reduced austenitic phase, thus substantiating the hardening effect of EDM.

#### Hollow NiTi wire mesh

The SAF file is also called an adaptive file and is designed as a compressible, thin-walled, pointed, hollow cylinder composed of a 120-mm-thick NiTi lattice. It has an asymmetrical tapered tip, a rough surface with 3-µm peak-bottom dimensions of 1.5 or 2.0 mm, 21, 25, or 31 mm, and the characteristics of continuous shape changes due to self-adjustment.

### Metallurgical development of NiTi alloy

With the continuous development of metallurgical technology, designers and manufacturers have improved the physical properties of metals, increased fatigue resistance by changing the alloy chemical and crystal phase composition. Thermal treatment, the most frequently used metal processing method, is applied to heat and cool a certain material under specific conditions several times to obtain a specific property of the material, such as the SE and SME of the NiTi alloy.

#### M-wire

In 2007, Tulsa Dental developed a new type of NiTi alloy, M-wire, after heat treatment of a conventional austenitic NiTi alloy. Under complex temperature conditions, a conventional NiTi alloy with austenite as its main crystal phase is prepared by a special stretching treatment, and the crystal phase transition temperature increases from 16–31 °C for the conventional NiTi wire to 47 °C. As a result, the alloy contains a large amount of stable martensite at room temperature, with a reduced stiffness and increased elasticity. In 2007, M-wire NiTi files represented by GT Series X and ProFile Vortex were developed. NiTi instruments composed of M-wire alloys, such as the ProTaper Next system and the ProFile Vortex system, have improved flexibility, fatigue resistance, and fracture resistance. Compared with the conventional NiTi alloy, the M-wire instrument has better centric positioning ability, can better maintain the original shape of the curved root canal, and can reduce canal deviation.

#### R-phase

SybronEndo successively introduced TF (2008) and K3XF (2011) (SybronEndo, Orange, CA) R-phase NiTi systems, in which conventional austenitic NiTi alloy is converted into R-phase NiTi alloy after multiple heat treatments. The Young’s modulus of the R-phase NiTi alloy is lower than those of alloys in the martensite and austenite phases, and more austenite is transformed into martensite under stress, which improves the elasticity and fatigue resistance of the file. In addition, the twisting fabrication method used for stainless steel instruments, such as the K-file, was used for the first time for the TF system. The heated R-phase NiTi wire is twisted, which protects the crystal structure of the NiTi alloy from being damaged and avoids fracture, which often occurs during the twisting process of conventional NiTi alloys.^28^ The K3XF system undergoes post-manufacturing heat treatment after the machining process is completed to accommodate some of the internal stress caused by machining.

#### CM-wire

Compared with the nickel content of 54.5%–57% of conventional NiTi alloy, CM NiTi wire contain less nickel (52%) which improves the mechanical properties of the alloy was introduced in 2010. In addition, DS Dental performs post-manufacturing heat treatments on alloy SE508, which further increases the austenite phase transition temperature of the CM-wire to 55 °C such that the crystal structure at room temperature is dominated by martensite. In conclusion, CM wires have become metal alloys with controlled memory, improved fracture resistance, good flexibility, and resistance to cyclic fatigue. The CM-wire file can be prebend to conform to the curvature of the root canal.^29^ If the instrument undergoes deformation (such as uncoiling) after use, it can return to initial shape once heated such as autocaved and can be reused, i.e., the so-called controlled shape memory effect. HyFlex CM, TYPHOON (DS Dental, Johnson City, TN), and M3 systems (M3, United Dental, Shanghai, China) are representative instruments.

#### Blue wire and gold wire

The CM-wire alloy undergoes repeated heat and cooling treatments to form a titanium oxide layer, and the surface color of the alloy varies with the thickness of the titanium oxide layer. When the thickness is 60–80 nm, the surface color is blue, i.e., the CM-Blue wire; when the thickness is 100–140 nm, the surface color is golden, i.e., the CM-Gold wire.^30^ The titanium oxide layer compensates for the hardness lost during the processing of CM-wire alloy and increases the cutting efficiency and wear resistance.^31^ Representative instruments include the Vortex Blue system (Dentsply Sirona, US), the Sequence Rotary system, the Reciproc Blue system, and the ProTaper Gold system.

#### Max-wire

Max-wire alloy is developed as a result of alloy heat treatment. In the early stage of root canal preparation, max-wire alloy is in the martensite phase at a low room temperature. After introduced into the root canal, when the temperature is equal to or greater than 35 °C, the max-wire alloy can shift from the martensite to austenite and the instrument changes from a straight shape to a semi-circular shape due to the shape memory function of austenite. During eccentric rotation against the walls of the root canal, the contact area between the instrument and root canal wall increases, which plays the roles of beating and scraping the root canal wall, when combined with agitation of irrigation fluid in the root canal, thus promoting root canal cleaning. The representative instrument is the XP-endo Shaper family system (FKG Dentaire, La Chaux-de-fonds, Switzerland).

The above mentioned metallurgical improvements enhance the flexibility of engine-driven NiTi instruments, better maintain the shape of the curved root canal, and reduce canal transportation; moreover, these improvements enhance the fatigue resistance of the instrument, reduce file separation due to torsion and fatigue, and improve safety in clinical practice. Concurrently, clinicians are concerned that with increased flexibility of the file after heat treatment, the mechanical cutting efficiency of the file is weakened. For example, the ProTaper Universal F2 system has better flexibility and lower surface hardness after heat treatment at 600 °C, while the cutting efficiency in the 3-mm region of the root apex is significantly decreased.^32^ Improvements in other aspects, such as the geometric design and heat treatment process, can compensate for and increase the cutting efficiency. For example, the ProTaper Gold system (Dentsply Sirona, US) has been confirmed to have the same or higher transverse and axial cutting efficiency.^33^

### Patency file/mechanical glide path instruments

Root canal patency refers to a smooth, unobstructed channel from the root canal orifice to the apical stop, and it allows the subsequent access of larger instruments. A smooth root canal patency can reduce the occurrence of iatrogenic errors during root canal preparation. In root canal preparation, the establishment of a gliding path for calcified and/or narrow canals is the most difficult procedure and requires high technical skills. Small stainless steel hand instruments such as #6, #8, and #10 root canal files were commonly used to gradually unclog the root canal with the watch-winding technique. In 2012, rotary NiTi PathFiles was proposed consisting of three files with a standard .02 taper and tip diameters of 0.13, 0.16, and 0.19 mm, which facilitate the flexibility and resistance to cyclic and torsional fatigue of the files during root canal exploration.^34^ It is recommended that mechanical glide path instruments, such as PathFile (Dentsply Maillefer, Switzerland), ProGlider (ProGlider (Dentsply Maillefer, Switzerland), ScoutRaCe (FKG Dentaire, Switzerland), R-pilot (VDW, Munich, Germany), and G-Files (Micro-Mega, France), to pre-enlarge the canals and facilitate advancement of subsequent larger size rotary NiTi instrumnents should be used only after establishment of initial patency of the canals by hand with a #10 file.

The common feature of mechanical patency files is the small tip diameter and taper. For example, the ProGlider and PathFile system made by M-wires have great flexibility and flexural strength. After unclogging small canals with stainless steel files #6, #8, and #10, ProGlider file with tip diameter #16 and a taper of 0.02 to 0.085 is used to achieve a wider canal compared with by hand stainless steel file #15 (ISO standard). R-Pilot and WaveOne Gold Glider NiTi glide path files are used with a reciprocating motion and would provide excellent cyclic fatigue resistance.

## Motion of the NiTi files

The majority of motion mode of engine-driven NiTi instruments is 360° full rotation with optimal cutting efficiency. New proposed motion modes such as reciprocating and axial motion, attempt to reduce the risk of separation of instruments.

### Modes of motion

#### Continuous rotary motion

In most NiTi systems, the file is driven by electric motors and reduction contra-angle handpieces to continuously cut the root canal wall through full rotation including centric and eccentric rotary motion in the root canal.^22^ Instruments with centric rotary motion, such as the ProTaper Universal and K3 systems, have a high cutting efficiency. But they can easily bind in the dentin, bear high torque, and are prone to torsional failure. The cross-sectional design of the ProTaper Next and XP-endo Shaper instruments can perform eccentric or asymmetrical rotary motion with a greater contact surface of the instrument with the canal and more efficiency at removing debris.

#### Reciprocating motion

In 2008, Yared proposed the concept of reciprocating motion of engine-driven NiTi system based on a balanced-force technique,^35^ that is, the root canal is prepared by different angles in the clockwise (CW) and counterclockwise (CCW) rotation. Many single-file NiTi instruments have been marketed, such as Reciproc, Reciproc Blue (VDW GmbH, Munich, Germany), Wave One, Wave One Gold, Pro Design R, Unicone, and X1 Blue File. Most reciprocating systems rotate with 120°–270° in CCW direction to cut dentin and remove debris by CW rotation with 60°–90°, which relieves torsion stress and intermittent buffering. Although some other systems, such as Genius and Pro Design S, cut the dentin by CW rotation.^22^ The Reciproc system is representative of using reciprocating motion, which performs CCW rotation at 150° and CW rotation at 30°. On this basis, the Reciproc blue CM system has a high resistance to cyclic fatigue and good flexibility after heat treatment. Another reciprocating motion instrument, the WaveOne Gold, performs CCW rotation at 170° and CW rotation at 50° and has four cutting edges with a rake angle of 85°. During the operation, only two cutting edges are in contact with each 200μm root canal wall circumference. Studies in vitro and in vivo have showed that re-use of reciprocating system can reduce the apical pressure of the instrument, prevent the taper lock phenomenon, provide lower incidence of torsional fracture and deformation when compared to continuous rotary motion.^22,36,37^

#### Axial motion

The characteristic of the SAF system is that the hollow file in the shape of a cylindrical meshwork without an internal core. The file operates in an in-and-out peking motion driven by a handpiece with frequency of 3 000–5 000 vibrations/min and amplitude 0.4 mm, with a combination of irrigation device to remove dentin by abrasion.^38,39^ In ovoid and irregular root canal space, the file can be stretched or compressed to adapt to the walls of the root canal.^40^

### Power-driven system

#### Handpiece

In 1892, Oltramare installed a fine needle with a square cross-section on an ordinary dental handpiece for endodontic treatment, creating the first rotary endodontic instrument in history.^41^ In 1899, Rollins developed the first special handpiece for root canal preparation, with a rotational speed of 100 rpm/min, and equipped it with a specially designed drill bit. The Giromatic system (MicroMega, France) was developed in clinical in 1964.^42^ With the development of rotary NiTi instruments, the electric handpieces and motor systems used with them are also undergoing constant development.

Early handpiece systems, such as the Giromatic, Endo-Gripper (Moyco Union Broach, Montgomeryville, PA, USA), Intra-Endo 3 LD (KaVo, Biberach, Germany), and DynaTrac instruments (Dentsply DeTrey, Konstanz, Germany), usually simulate the movement of manual instruments, i.e., alternative rotation of 90° in the CW and CCW directions. The handpieces of the M4 (SybronEndo, Orange, CA, USA), Endo-Eze (Ultradent Products Inc., South Jordan, UT, USA), and Endo-Express SafeSider instruments (Essential Dental Systems, South Hackensack, NJ, USA) are examples of reciprocating rotate equal 30° of CW and CCW rotation to prepare the root canal.^43^ However more procedural errors such as zip, elbow, ledges, and even perforation were produced in curved canals.^44^

Currently, low-speed electric handpieces with controlled torque setting are often used in practice, such as NSK reciprocating handpieces (Nakanishi, Tochigi-ken, Japan), Endo IT electromotors (VDW, Germany), and some new handpieces used for NiTi files that can perform axial movements (e.g., Canal Finder System: Societé Endo Technic, Zurich, Switzerland; Endolift: SybronEndo).^45^ The electric reduction motor requires a special match handpiece in gear size and final output.

#### Motors

The electric endodontic motor can be classified into three types, a continuous rotation motor with controlled torque, a reciprocating rotation motor, and an axial motion motor.

Continuous rotation motors are the most frequently used motors for engine-driven NiTi files with a preset speed and torque according to the manufacturer’s instructions. When the file rotates and cuts, if it encounters rotation resistance exceeding the preset value, then the motor rotate reversely automatically to allow the file loosen and withdraw from the root canal to reduce the torsional fracture. Studies have found that at low speeds, such as 150 rpm, less lock-in, deformation, and fracture of the file occur. While at high speeds, such as 250 and 350 r·min^-1^, the cleaning efficiency can be improved but the instrument is prone to lock-in.^46,47^ The torque generated by rotating the NiTi file during root canal preparation depends on the volume of the root canal, the pressure applied by the operator, the diameter of the instrument, the design of the cross-section, the number of reuses, the manufacture process, and the contact area between the instrument and the root canal wall.^48,49^

New electric hybrid motors were introduced to combine rotary and reciprocating movement. In 2012, Sybron Endo developed hybrid motor Elements, which can work with both kinematics depending on the pressure on the instrument during root canal preparation and used with TFA NiTi system.^22^ Genius system (Ultradent, South Jordan, UT, USA), which was introduced in 2016, has both CW rotation and reciprocating motion modes. During initial preparation, reciprocating motion is used to allow safer canal negotiation; then CW rotation mode is used to provide efficacy of instrumentation. Studies have confirmed that in double-curved root canal models, the Genius system has a higher torsional resistance than the reciprocating single-file system.^22^

Subsequently, the optimum torque reverse (OTR) motor (Tri Auto ZX2 J Morita, Kyoto, Japan) was designed in Japan, which regulates the motion mode by monitoring the torque of the instrument. When the torque is lower than the preset value (trigger torque), the motor drives the instrument to continuously rotate CW. When the torque is larger than the preset value, the motor automatically drives the instrument to rotate with CCW by 90° and then continues to rotate with CW by 180° (ref. ^50^). This motion is repeated until the torque is lower than the preset value. This design canrelease the torque by asymmetric oscillatory motion in the opposite direction of the cutting, thereby reducing the risk of excessive stress and increasing the fatigue resistance of the instrument.^51^ The OTR motor can also be used with NiTi instruments with CW rotation, such as the PTN, Revo-S, Mtwo, TF, and EndoWave systems.^52^

To match the motion mode used by the SAF system, the electric motor can also drive the file to move up and down to facilitate cleaning of noncircular sections of the root canal, and this type of motor is known as an axial motor.

In addition, according to the preset parameters, electric motors can be classified as open or closed motors. For open motors including the ATR Vision (ATR, Pistoia, Italy), iEndo Dual (Acteon, Merignac, France), and SAF Pro System (ReDent-Nova, Ra’anana, Israel), parameters such as speed, rotation direction, and angle, while the parameters of closed motors, such as the WaveOne, Reciproc, Elements moto (SybronEndo, Orange, CA, USA), and ATR Technika (ATR), are preset and cannot be changed.

In summary, with the advancements of technique in geometric design, manufacturing, thermal treatment, metallurgy, a wide variety of engine-driven NiTi systems have been developed and can provide better shaping efficiency and resistance to cyclic and fatigue.

Changes of morphological design and manufacturing process on NiTi instruments have improved the cutting efficiency; Shifting of crystal phase of the material has increased fatigue resistance and reduced fatigue fracture; and the variety of motion mode has improved resistance to torsional failure. With improvements in the alloy material and manufacture process by the designers, the flexibility of the file and the resistance to cyclic fatigue and torsional failure have continuously improved. Careful consideration should be given to compare a NiTi system with another directly in cutting efficiency and safety, which are affected by some factors not only the design of files but also the clinical situation. In clinical practice, an appropriate system should be selected based on the anatomy of the root canal, instrument characteristics, and operators’ experience.
